# Influence of job environment on the online teaching anxiety of college teachers in the online teaching context: The mediating role of subjective well-being

**DOI:** 10.3389/fpubh.2022.978094

**Published:** 2022-10-14

**Authors:** Xu Zhang, Suqi Li, Shuwen Wang, Jinlei Xu

**Affiliations:** ^1^Faculty Development Center, Nanjing University of Posts and Telecommunications, Nanjing, China; ^2^School of Education Science, Nanjing Normal University, Nanjing, China

**Keywords:** teaching anxiety, online teaching, job demands, job resources, subjective well-being

## Abstract

**Background:**

Online education has been conducted widely in higher education in recent years. While online teaching brings many opportunities, it also poses numerous challenges and issues. This is especially true for college teachers, for whom teaching is considered to be a profession with a high level of burnout and anxiety. The large-scale application of online teaching methods has put teachers in an even more challenging context, which may lead to teaching anxiety affecting their mental health. In online teaching contexts, the question of what factors affect college teachers' online teaching anxiety is worth exploring to help reduce their online teaching anxiety so as to promote their work performance. In this study, therefore, we conducted a survey of college teachers to develop a model of job environment (job demands and job resources), subjective well-being, and online teaching anxiety, and to explore the influences of job environment and subjective well-being on their online teaching anxiety, as well as the mediating effects of subjective well-being between job environments and online teaching anxiety.

**Method:**

Of the 1,060 college teachers who participated, 524 were male (49.4%) and 536 were female (50.6%). An online questionnaire was sent to the teachers in January, 2022. Online teaching anxiety, subjective well-being, and job environment scales were adapted and developed. Descriptive analysis, reliability and validity analysis, and structural equation modelling were used to analyse the collected data.

**Results:**

The study model showed an adequate fit (χ^2^ = 440.983, RMSEA = 0.070, GFI = 0.942, AGFI = 0.914, NFI = 0.949, and CFI = 0.956), confirming the relationships of job demands and online teaching anxiety (β = 0.310, *p* < 0.001), job resources and online teaching anxiety (β = – 0.086, *p* < 0.01), job demands and subjective well-being (β = – 0.411, *p* < 0.001), job resources and subjective well-being (β = 0.204, *p* < 0.001), and subjective well-being and online teaching anxiety (β = – 0.435, *p* < 0.001). Meanwhile, the results also proved the effects of the mediating role of subjective well-being between job demands (95% CI = [– 0.138, – 0.225]), job resources (95% CI = [– 0.119, – 0.064]), and online teaching anxiety. The model accounted for 33.8% (*f*^2^ = 0.401) of online teaching anxiety.

**Conclusion:**

The results of this study indicated that it is important to reduce job demands and increase job resources to alleviate college teachers' online teaching anxiety to maintain good mental health; while maintaining a high level of college teachers' subjective well-being is also helpful for promoting their work performance. Furthermore, the indirect effects of job demands and job resources on online teaching anxiety mediated by college teachers' subjective well-being were also significant.

## Introduction

The rapid development of educational informatization has provided a broader scope for online education (1). In recent years, online teaching has been conducted at all educational stages, and higher education is no exception (2). Compared to traditional offline teaching, there are some significant advantages to online teaching (3). For example, conducting online teaching mainly depends on good network connections and synchronous online teaching platforms such as Tencent Meeting or Zoom, which break though the boundaries of geography and time (4), putting learners in a more flexible learning environment (5). The widespread conduct of online instruction makes it possible to share high-quality educational resources, which used to be restricted to specific regions or schools (6). In addition, digital competencies and other information and education technologies are what teachers need to be equipped with in the industry 4.0/5.0 era (7). Some studies have pointed out that online instruction provides teachers with opportunities to develop their digital competence and teaching resilience (8). Overall, whether it is due to the openness or inclusiveness of online teaching (9), it offers many advantages that are difficult to achieve with traditional face-to-face teaching.

Online teaching has, however, also brought many challenges for teachers, such as unstable networks (10), the limitation of technology literacy (11), and the poor interaction between teachers and students (12). A delay in image or video transmission could be caused due to an unstable network (13). Gao and Zhang (14) pointed out that it was hard for some teachers to carry out effective online instruction due to their lack of ability to use technology. Some teachers had little experience of online teaching (15), and limited online technological training (16), which might lead to the decrease in interaction effectiveness between teachers and students (17). In addition, because “face to face” teaching was replaced by “face to computer” teaching, the teaching activities and teaching objectives need to be redesigned to adapt to online learning contexts (18), which would undoubtedly add to teachers' burnout due to the time and effort required. Furthermore, for college teachers, some practical courses exist in universities. In the context of online teaching, these courses are more difficult to implement (19), which can also cause teaching anxiety for teachers. In fact, teaching has always been seen as a profession full of stress and anxiety (20), even in the traditional face-to-face mode of teaching. What factors affect teachers' teaching anxiety in traditional teaching models has been the focus of research by a number of researchers. Klassen and Chiu (21) found that teachers' teaching anxiety would be influenced by some demographic factors such as gender, age, years of teaching experience, personality traits, and so on. Han et al. (22) applied the job demands-resources (JD-R) model proposed by Demerouti et al. (23) to explore the relationship between job demands-resources and teaching anxiety among university teachers, and the results showed that a high level of job demands could aggravate teachers' teaching anxiety, whilst these impacts caused by high job demands could be diminished by job resources (24). Furthermore, teaching is “an emotion-laden process” (25), and some studies have also been carried out to explore the impact of teachers' mental health status on teaching anxiety (26). Dewaele and Mercer (27) found that teachers' psychology played a vital role in the quality of teaching. Nazari and Oghyanous (28) conducted a study to explore the relationship between the psychological well-being and stress of teachers, and found a significant relationship.

In the online teaching context, teachers will face more challenges and anxieties than in the traditional offline teaching model (29), so is it possible that some of the factors that influence teachers' anxiety in the traditional teaching model are still present in online teaching? To answer this question, this study aimed to explore the relationships between college teachers' online teaching anxiety, job environment, and subjective well-being to help teachers maintain good mental health and achieve high-quality work performance.

## Theoretical background and hypotheses

### Online teaching anxiety

Teaching anxiety refers to an emotion of panic or tension that teachers in a specific situation experience; it is caused by pressure or a changing environment (30). Teaching has been widely considered as an anxiety-inducing profession (20). In traditional instruction, there are many factors that lead to the generation of teachers' teaching anxiety. For example, teachers would feel anxious when they prepare or organize teaching activities before or during classes (31). The lack of control over the teaching environment and teaching events can also add to teachers' teaching anxiety (32). Additionally, with the development of Informational Computer Technology (ICT), it could be a little difficult for some teachers to have adequate mastery of manipulating modern technology-driven teaching equipment (33), which also adds to teachers' anxiety.

In the online context, teachers' online teaching anxiety still exists, and compared to previous teaching anxiety, online teaching anxiety has a more significant impact on teaching effectiveness (34). Firstly, the frequency of the occurrence of technical problems in online teaching contexts is higher than in face-to-face instruction (35), such as the delay in transmission (sounds, picture, video, or other instructional materials) (36), and teachers' unfamiliarity with some online teaching devices or platforms. Those technological issues will bring additional teaching anxiety for teachers (37). Furthermore, the interaction between teachers and students also poses difficulties. In the online teaching context, due to the technical restraints, the pictures of teachers and students are limited to the area shown by the screen, and thus, non-verbal communication (such as facial expressions or behavioral language) may be less apparent than in face-to-face classrooms. Such limitations may lead to weak interaction between teachers and students (38), which could lead to a high level of teaching anxiety.

### Subjective well-being (SWB)

Well-being refers to a multidimensional construct including subjective, psychological, and social well-being (39). Among them, subjective well-being focuses on the presence of positive emotion and satisfaction, as well as the absence of negative emotion (40). The level of teachers' subjective well-being is often measured according to their subjective evaluation of their lives and work (41). It can indicate the level of well-being of people according to their subjective evaluation of their lives and work (41). Expanding on the concept of teachers' subjective well-being, it refers to teachers' evaluation of their professional lives both inside and outside of schools (42). Some measures have been developed to evaluate teachers' subjective well-being, including the positive and negative affect schedule (PANAS) (43) and a teacher subjective well-being questionnaire (TSWQ) (44). In previous studies on subjective well-being, researchers have pointed out that teachers' subjective well-being is influenced by many factors (45) such as self-efficacy (46), emotional intelligence (47), work environment (48), and work engagement (49). Furthermore, because of the complexity of teaching (50), teaching used to be seen as a highly emotional profession that comes with challenges and stress (51). However, some studies have indicated that teachers' subjective well-being was negatively related to job burnout, anxiety, and depression (52). In other words, when teachers have a high level of subjective well-being, their negative emotions and stress will be alleviated.

Generally, teachers with high SWB are likely to be successful in their work, love, and performances (53). Especially in online teaching contexts, it is even more necessary for teachers to maintain a high level of SWB.

### Job environment

The job demands-resources (JD-R) model was proposed by Bakker and Demerouti (54). The model includes two categories (job demands and job resources). Some previous studies have explained the model as follows: every job has specific components related to job stress, and job stress will increase when job demands are high and when job resources are limited (55). Ideally, a good job environment should provide workers with sufficient job resources to fulfill their job demands, and keep their job demands at a relatively low level, so as to mitigate their job stress or anxiety. However, in the actual job environment, teachers are provided with job resources that often do not meet the job demands. It is therefore worth using the JD-R model to evaluate teachers' actual job environment. Following are more specific explications of the two categories (job demands and job resources).

#### Job demands

Job demands refer to the efforts put in by employees in terms of physical, psychological, social, or organizational aspects (23). Naidoo-Chetty and Plessis (56) divided job demands into three aspects, namely quantitative demands, qualitative demands, and organizational demands. Generally, job demands include job complexity, emotions, high workload, time pressure, and low student motivation (57–59). For college teachers, all descriptions of job demands mentioned above have some influences on them. For example, many studies have indicated that job demands could lead to the generation of job burnout (60). With the increase in job demands, more of teachers' energy will be consumed to cope with these job demands (23), and teachers will reduce their efforts to restore loss or obtain new resources (61). Previous studies pointed out that high job demands had a negative influence on psychological stress (62), and made the job objective highly challenging (63). Han et al. (22) also indicated that a high level of job demands can lead to stress, emotional exhaustion, and decreased satisfaction. When teachers are in a high demand job environment for long periods of time, their teaching outcomes, job satisfaction, physical health, and well-being are likely to be negatively affected (64).

In online teaching contexts, college teachers will be faced with more challenges and anxiety caused by job demands (29). What should we do to reduce college teachers' teaching anxiety through the perspective of job demands? This study attempted to settle this question by exploring the relationship between job demands and online teaching anxiety.

#### Job resources

Job resources refer to aspects of the job that can help individuals effectively cope with strain and help reduce the psychological and physical costs associated with the job demands (65). Schaufeli (66) suggested that job resources include two aspects, namely organizational resources and personal resources. In van Rensburg's (67) study, job resources included job satisfaction, motivation, job engagement, and job performance. Additionally, Bakker and Demerouti (54) proposed that job resources should include the following four levels: organization (e.g., security, job opportunities); interpersonal and social relations (e.g., the supports from colleagues and leaders); job organization (e.g., role clarity, engagement); and tasks (e.g., autonomy, feedback, task identify). In the current study, the job resources of college teachers at the social and teaching level were evaluated. Many studies have been conducted to confirm the influence of job resources on teachers. It was found by van Woerkem et al. (24) that job resources can diminish the damage caused by a high level of job demands and attenuate the consumption of resources. The social support from colleagues and students could alleviate teachers' workload. Meanwhile, job resources have a positive influence on teachers' positive teaching performance and well-being (68).

Especially in online teaching contexts, it is worth providing college teachers with sufficient job resources to diminish the negative impacts caused by job demands. Therefore, one of the aims of this study is to explore the relationship between job resources and online teaching anxiety.

### Research model and hypotheses

**Figure 1:**
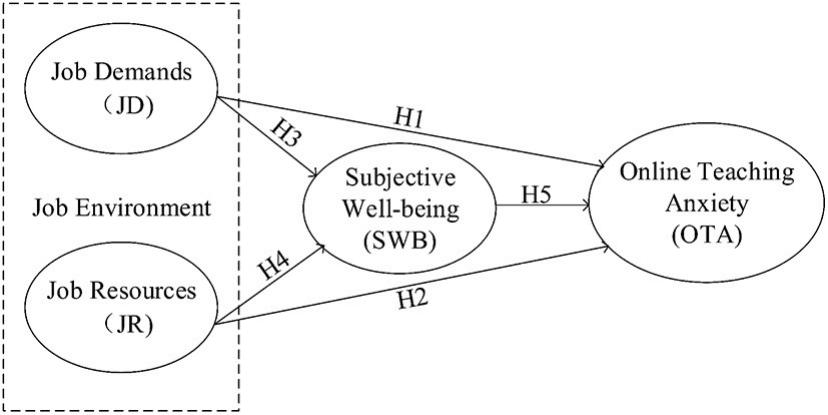
Hypothesized model.

According to the above literature review, we have learnt that in traditional face-to-face teaching environments, teachers' teaching anxiety could be influenced by their job demands, job resources, and their well-being; whether these factors would have any influence on college teachers' online teaching anxiety in the online teaching context is still unknown. Thus, the current study aimed to develop a hypothesis model to explore the relationships between those factors in online teaching contexts (see Figure 1).

Teaching has been considered to be one of the high-demand jobs (69). The job demands-resources (JD-R) model has been widely used to interpret burnout (70). Although developed to explain burnout in the context of paid work, the model can be applied to other contexts including universities, where “job demands and resources” can be read as “task” or “role” demands and resources. Central to the model is the idea that job demands are factors or tasks that require sustained physical or mental effort to complete (23). This is not necessarily problematic for an individual unless the costs exceed their adaptive capacities, in which case they can lead to exhaustion and burnout. Job resources are physical, psychological, social, and organizational factors that reduce the costs associated with job demands, are functional in achieving work goals, and stimulate personal growth, development, and learning. Now, in the online teaching context, it is even more challenging for college teachers to work in such a job environment, and many teachers are usually more anxious than when teaching offline (29). To maintain the same teaching effectiveness as in the traditional face-to face teaching mode, college teachers need to meet more job demands, such as good network access, proficiency in the use of teaching platforms, and maintaining good interaction with students (71). Consequently, more job resources should be provided to college teachers. In that case, it is worth exploring the relationship between college teachers' job environment (job demands and job resources) and their online teaching anxiety. The following hypotheses were therefore proposed in this study:

H1. Job demands are positively related to online teaching anxiety.
H2. Job resources are negatively related to online teaching anxiety.

In addition, teachers' subjective well-being refers to the subjective evaluation of teachers' lives and works (41). Previous studies have indicated that school factors are associated with teacher subjective well-being (72). Teachers have been found to have subjective perceptions of their job environment (55). Generally, this job environment includes both the physical environment, such as the cleanliness of the classrooms and campus, the adequacy of the teaching facilities, and the psychological perceptions that teachers perceive in their working environment, such as the friendliness of their colleagues, the positive interaction with their students, and the support from their leaders (68). However, in the online teaching environment, the physical environment has changed significantly, and the connection between teachers and their colleagues and students has become weaker (73). Does the job environment (Job demands and Job resources) still affect teachers' subjective well-being in this context? The current study attempted to answer this question. The following hypotheses were proposed:

H3. Job demands are negatively related to subjective well-being.
H4. Job resources are positively related to subjective well-being.

Furthermore, based on the above literature review we learnt that teachers' psychological state would affect the level of their anxiety. Some studies showed that teachers' psychological state during online teaching cannot be ignored. When teachers' teaching satisfaction is high, their job anxiety is also relieved. It is therefore helpful to alleviate teachers' teaching anxiety by maintaining a high level of teaching satisfaction (73). Subjective well-being is comprehensive in nature, and one of its dimensions is satisfaction, thereby there may also be a relationship between subjective well-being and anxiety (74). What's more, previous studies have indicated that the more anxious teachers feel, the lower their level of subjective well-being. On the contrary, high subjective well-being can predict one's positive level of mental health states (75). The degree of teachers' teaching anxiety also affects teachers' mental health. Therefore, this study considered that teachers' subjective well-being would also influence their online learning anxiety. The following hypothesis was thus proposed:

H5. Subjective well-being is negatively related to online teaching anxiety.

Subjective well-being reflects the level of well-being of people according to their subjective evaluation of their lives and work (41). For teachers, the higher level of teachers' subjective well-being, the more satisfied they are with their teaching performance; therefore, the anxiety present in their daily teaching becomes weaker (76). In addition, when teachers are in a good job environment, that is, when they are provided with sufficient job resources but have low job demands, their teaching satisfaction is enhanced and their anxiety tends to be alleviated. Furthermore, the definition of subjective well-being revealed that it is a multidimensional concept, and the degree of teaching satisfaction can reflect the level of teachers' subjective well-being (50). Thus, job resources and job demands can directly affect teachers' teaching anxiety, and can also indirectly affect teaching anxiety through their subjective well-being. In the context of online teaching, teachers' teaching anxiety still remains, and can even be exacerbated, due to changes in teaching styles. Is it still effective to keep teachers' subjective well-being high in order to alleviate their anxiety in the online context? The following hypotheses were proposed to address this question:

H6. Subjective well-being acts as a mediator between job demands and online teaching anxiety.
H7. Subjective well-being acts as a mediator between job resources and online teaching anxiety.

## Methodology

### Participants and data collection

In the current study, simple random sampling was adopted and the online questionnaire was uploaded on Questionnaire *Star* (www.wjx.cn) (accessed on 22 January 2022), a professional online survey tool widely used in China (77). The link to the online questionnaire was sent to the department of the teacher professional development of some colleges in Jiangsu, China. Then the department staff sent the link to their teachers. In the first part of the questionnaire, participants were told that they were participating in an anonymous study, the content of which may be published without any commercial use. If they did not want to participate in the survey, they could quit the website of the online questionnaire. Participants who agreed to participate in the survey filled in the questionnaire. In addition, to ensure the reliability of the results of the subsequent data analysis, the ratio of the number of questionnaire items to the number of participants should be 1:5, and the larger the sample size the better (78). Therefore, eventually, 1076 teachers agreed to took part in the study by completing the online questionnaire. After deleting those questionnaires with the same answer for all items or overly short response time, the effective number of samples was 1,060, with an effective rate of 98.5%. Table 1 shows the demographic data from the survey participants, such as gender, age, teaching age, educational level, job title, and subject.

**Table 1:** Socio-demographic profile of respondents.

**Sociodemographic**	**Characteristics**	***N***	***%***
Gender	Male	524	49.4
	Female	536	50.6
Age	<25 years	33	3.1
	26–30 years	91	8.6
	31–35 years	317	29.9
	36–40 years	182	17.2
	41–45 years	217	20.5
	>46 years	220	20.8
Teaching age	1–3 years	325	30.7
	4–6 years	149	14.1
	7–10 years	117	11.0
	>11 years	469	44.2
Educational level	3-year college and below	6	0.60
	4-year college or university	106	10
	Master degree	551	52.0
	Doctor degree	397	37.5
Professional title	Assistant professor	83	7.8
	University lecturer	561	52.9
	Associate professor	315	29.7
	Professor	101	9.5
Subject	Liberal arts	372	35.1
	Science	216	20.4
	Engineering	394	37.2
	Arts	43	4.1
	Sports	35	3.3

#### Instrument

The online questionnaire consisted of three parts. In the first part, we stated that this survey was being conducted voluntarily and anonymously. The answers to the questionnaire were only available for the researchers and not for commercial or any other use. The second part was to collect the participants' basic information. The third part was a scale designed for the variables in the study. The scale of the questionnaire used in this study was adapted from previous studies. The original scale was developed in English; thus, all items were translated into Chinese following the translation-back-translation procedure (79). To ensure semantic equivalence, two Chinese bilingual academics translated all items into Chinese separately and individually translated them back into English. Two experts reviewed all items to ensure face validity. In addition, to ensure the readability of all items, three college teachers were invited to read all items and give feedback. Finally, according to Zhan et al. (80), some volunteers were asked to answer the questionnaire. It was found that the questionnaire should be finished in at least 2 minutes. Therefore, if the response time of a questionnaire was less than 2 minutes, it was considered as a short response and was excluded from the analysis.

#### Online teaching anxiety scale

The dependent variable of the current study was online teaching anxiety. The items of the scale were adapted and developed by Bilali (81) to cater to the context of online teaching. The final scale included six questions (e.g., During online teaching, I am worried that I will not be able to maintain the online classroom well.). A 5-point Likert scale was used to evaluate the degrees of college teachers' online teaching anxiety from strongly disagree to strongly agree as 1–5. The higher the score, the higher the degree of teaching anxiety, and vice versa.

#### Subjective well-being scale

The scale of subjective well-being was developed by Renshaw (44). For this study we adapted it for college teachers according to the online context. Finally, seven items were applied in the study. As above, a 5-point Likert scale was also used to assess college teachers' subjective well-being, and the higher their score, the higher their subjective well-being.

#### Job environment scale

In the current study, job environment included two sub-dimensions: job demands and job resources. The assessment items of college teachers' job demands and resources originated from Podsakoff et al. (70, 82). The specific description of items used in the current study was modified according to the college teachers' online teaching context. There were five items for job demands, and five for job resources. For the dimension of job demands, it was evaluated through the two sub-dimensions, that is, time management (e.g., During online teaching, I rarely have enough time to do everything), and classroom organization (e.g., During online teaching, it's hard for me to ensure that my students focus on their learning tasks). In terms of job resources, it included both organization and interpersonal relationship sub-dimensions; for example, “During online teaching, I place great importance on maintaining good communication with students”. A 5-point Likert scale was also used, and the higher their score, the higher their job demands and the more satisfied they were with the job resources.

#### Reliability and validity analysis

Construct reliability, convergent validity, and discriminant validity tests were conducted to examine the reliability and validity of the hypothetical model (83).

First, SPSS 21 was used to conduct exploratory factor analysis (EFA), and items with factor loading values less than 0.5 in each construct were deleted from each construct. Then, confirmatory factor analysis (CFA) was conducted to the reliability of instrument, and items with the highest residual value in each construct were deleted until the CFA values reached the threshold suggested by Hair et al. (84). The measurement model exhibited a good fit, with χ^2^ = 283.977, *df* = 71, *p* < 0.001, χ^2^/*df* = 4.000, GFI = 0.926, NFI = 0.942, CFI = 0.956, and RMSEA = 0.075. Hence, 14 remaining items were kept for further analysis, including three items each for job demands and job resources, and four each for subjective well-being and online teaching anxiety.

Then, composite reliability (CR) and Cronbach's alpha were applied to measure the reliability of the instrument (84). Construct reliability reflected the degree to which the items consistently demonstrated the latent construct (85). The rule of thumb is that both reliabilities need to be above the 0.70 minimum threshold (86, 87). In the current study, the criteria of CR and Cronbach's α were both higher than 0.70, which indicated that good composite reliability is present in this study (see Table 2).

**Table 2:** Reliability results and AVE results of constructs.

**Constructs**	**FL**	**CR**	**AVE**	**Cronbach's Alpha**
Job demands	0.780–0.886	0.887	0.724	0.880
Job resources	0.836–0.908	0.914	0.780	0.897
Subjective well-being	0.663–0.859	0.858	0.604	0.826
Online teaching anxiety	0.757–0.884	0.896	0.684	0.878

CR, composite reliability; AVE, average variance extracted.

In addition, the value of average variance extracted (AVE) was calculated to check the validity of the measurement model. According to Fornell and Larcker (86), the value of AVE should be above 0.50. In this study, as shown in Table 2, the AVE values of all variables exceeded the recommended 0.50, ranging from 0.604 to 0.780, which indicated that the measurement model was effective.

Furthermore, discriminant validity is usually evaluated through the square root of AVE. The discriminant validity was assessed by assuming that the AVE's square roots are higher than the construct's highest squared correlations with other latent constructs. The AVE's square root can be used to assess discriminant validity in each latent variable if this value is greater than other correlation values. Table 3 shows that the AVE's square root for each construct surpassed similarities between the construct and the other constructs, and so met the criterion of discriminant validity.

**Table 3:** Discriminant validity.

**Constructs**	**Job demands**	**Job resources**	**Subjective well-being**	**Online teaching anxiety**
Job demands	**0.851**			
Job resources	– 0.105*	**0.883**		
Subjective Well-being	−0.472***	0.194***	**0.777**	
Online teaching anxiety	0.546***	– 0.233***	– 0.619***	**0.827**

^***^ Significant at the 0.001 level; ^*^Significant at the 0.05 level. The bold values are the square root of AVE.

Overall, the results indicate that all values were higher than the minimum benchmark. Thus, the reliability and validity of the current measurement model were successfully demonstrated.

#### Common method bias

Common method bias was tested using Harman's single-factor test, and an exploratory factor analysis was run to ensure the reliability and validity of all items. The results indicated that the eigenvalues of four factors were all greater than 1, and the variation explained by the first factor accounted for 21.386%, which is less than the recommended threshold (50%) (82). By extrapolation, there was no problem of common method bias in this study.

#### Data analysis

In this study, SPSS 21 and AMOS 24.0 were used to test the structural equation model. First, SPSS was used to conduct Exploratory Factor Analysis (EFA) to initially determine the fit of the observed data. Then AMOS 24.0 was applied to conduct the construct reliability, convergent validity, and discriminant validity tests to examine the measurement model. Next, the goodness of fit of the structural model was assessed through the Confirmatory Factor Analysis (CFA), and the goodness of fit was measured by the likelihood ratio chi-square (χ^2^), GFI, AGFI, TLI, CFI, and RMSEA. Finally, AMOS 24.0 was employed to conduct the path analysis to examine the research hypotheses of the structural model.

## Results

### Model fit analysis

SEM analysis was applied to test the hypothesized structural model. Prior to the assessment of the structural model, in order to guarantee that the hypothesized relationships were correct, the goodness of fit of the structural model was assessed. As shown in Table 4, all indices were within the acceptable ranges, which indicated that the hypothesized structural model was reasonable, χ^2^ = 440.983, RMSEA = 0.070, GFI = 0.942, RMR = 0.058, AGFI = 0.914, NFI = 0.949, and CFI = 0.956.

**Table 4:** Fit measures for the structural model.

**Measure**	**Threshold**	**Value**
Chi-square (χ^2^)	*p* > 0.01	440.983
Root mean square of error approximation (RMSEA)	<0.08	0.070
Goodness-of-fit index (GFI)	>0.90	0.942
Root mean square residual (RMR)	<0.08	0.058
Adjusted goodness of fit index (AGFI)	>0.90	0.914
Normed fit index (NFI)	>0.90	0.949
Comparative fit index (CFI)	>0.90	0.956

#### Path analysis

**Figure 2:**
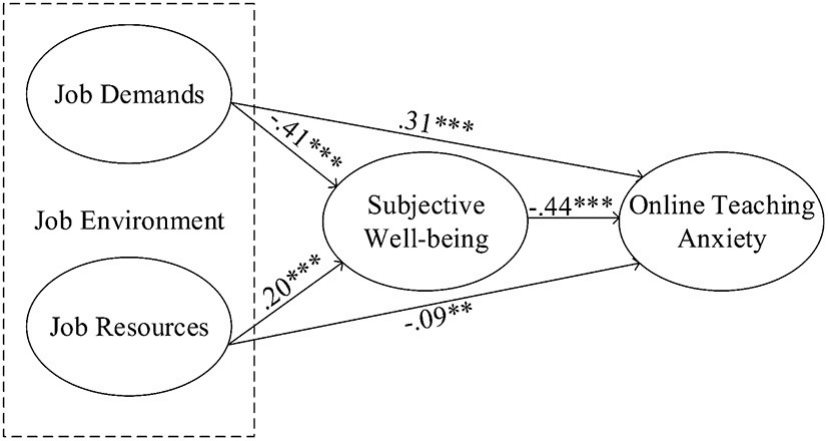
Path analysis results.

In order to confirm the five hypotheses, AMOS 24.0 was applied to calculate the correlation among the four latent constructs and the research model's explanatory power. The hypothesis testing results are reported in Table 5 and Figure 2. The results indicated that the five hypotheses proposed above were all significantly supported. Specifically, hypothesis 1 predicts a positive relationship between job demands and online teaching anxiety, and it was accepted at (*p* = 0.000). H2 means the job resources are negatively related to online teaching anxiety, and it was accepted at (*p* = 0.003). H3, which predicts that a positive relationship between job demands and subjective well-being, was accepted at *p* = 0.000. Finally, the negative relationship between job resources and subjective well-being (H4) was also accepted at *p* = 0.000. The negative relationship between subjective well-being and online teaching anxiety (H5) was also supported at *p* = 0.000. Overall, the sizes of the structural coefficients for the supported hypotheses were all considered meaningful for interpretation purposes.

**Table 5:** Hypotheses testing results.

**Hypotheses**	**Hypotheses paths**	**Estimate**	**SE**	**CR**	***p*-values**	**Supported**
H1	JD → OTA	0.310	0.031	9.414	0.000***	Yes
H2	JR → OTA	– 0.086	0.042	– 2.980	0.003**	Yes
H5	SWB → OTA	– 0.435	0.032	– 12.051	0.000***	Yes
H3	JD → SWB	– 0.411	0.035	– 12.389	0.000***	Yes
H4	JR → SWB	0.204	0.053	6.336	0.000***	Yes

^***^ Significant at the 0.001 level; ^**^ Significant at the 0.01 level.

The coefficient of determination (R^2^) represents the overall impact of the exogenous variable on the endogenous variable. R^2^ values higher than 0.6 are considered to have a high impact effect, 0.3–0.6 are considered medium, and less than 0.3 is considered as having a low impact effect (88). Those R^2^ values in Figure 2 indicate that JD and JR had a low impact on SWB, and the effect of JD, JR, and SWB on OTA was medium. In addition, effect size (Cohen's *f*^2^) was proposed by Cohen, where *f*^2^ values greater than 0.8, between 0.2 and 0.8, and less than 0.2 can be considered as large, medium, and small, respectively (89). For the model, the explanatory power of JD, JR, and SWB on OTA was 33.8% (*f*^2^ = 0.401). Hence, the five variables in this study have good predictive power (90).

#### Indirect effects of JD and JR on OTA mediated by SWB

To provide additional evidence to explore whether the indirect effects contained in the research model are significant, 1,000 resample bootstrapping was performed in this study. The bootstrapping results are shown in Table 6, which provides the standardized coefficient and upper and lower bound of 95% confidence intervals. It can be observed that the bootstrapping confidence intervals of indirect effects did not comprise zero in the two paths, including JD → SWB → OTA (95% CI = [0.138, 0.225]) and JR → SWB → OTA (95%CI = [– 0.119, – 0.064]). Therefore, JD was positively related to OTA mediated by SWB, revealing that H6 was supported; and JR was negatively related to OTA mediated by SWB, revealing that H7 was supported.

**Table 6:** Bootstrapping results.

**Model paths**	**Standardized coefficient**	**95% CI**	
		**Lower bound**	**Upper bound**
Indirect effect			
JD → SWB → OTA	0.179	0.138	0.225
JR → SWB → OTA	– 0.089	– 0.119	– 0.064

## Discussion

The current study was carried out to explore the influence of job environment (job demands and job resources) and subjective well-being on college teachers' online teaching anxiety in the online teaching context. Based on previous studies, a hypothetical model was constructed. The model identified some variables to predict college teachers' online teaching anxiety. In general, according to the results, good fit values were acquired by the model, and the five hypotheses were all supported. Following are specific explanations of the results.

### The influence of job environment on college teachers' online teaching anxiety

According to the results, the hypotheses that job demands are positively related to online teaching anxiety (H1) while job resources are negatively related to online teaching anxiety (H2) were both supported. On the one hand, teachers' teaching anxiety was a part of teachers' job stress (87). Previous studies have indicated that the management of students' classroom behavior, as the main job demand, leads to teachers' job stress (91). Especially in the online teaching context, compared to face-to-face teaching, it was more difficult for teachers to observe in real time the classroom behaviors of each student through the computer or other display, which certainly added more resistance to effectively managing the order of online classrooms. Therefore, teachers' online teaching anxiety was also magnified. In addition, college teachers were also under the pressure of time. Due to the change of job environment for teachers, they had to re-design the class routines and teaching content according to the online teaching context (92), which certainly added to their job load, and they need to spend more work time tackling those problems. On the other hand, according to the results, the job resources, and mainly emotional resources (93), provided by schools, colleagues, students, and so on will in turn mitigate college teachers' online teaching anxiety; thus, there is a negative relationship between job resources and online teaching anxiety. For college teachers, a good relationship with colleagues will alleviate their teaching anxiety (94). This influence persists in the context of online teaching, where the better the relationship between teachers and their colleagues, the less teaching anxiety teachers will feel. In addition, it is vital for teachers to maintain good interaction with students (95). A positive teacher-student relationship could motivate students to more actively participate in the classroom learning, and thus acquire good achievements for students as well as high quality teaching practices for teachers (96); this can in turn mitigate teachers' teaching anxiety. Especially in the online teaching context, teachers are isolated for a long time (97), so a good relationship with colleagues and students can be all the more valuable.

#### The influence of job environment on the subjective well-being of college teachers

This study also explored the impact of job environment on college teachers' subjective well-being, where the job environment included two parts: job demands and job resources. According to the results, the hypotheses that job demands are negatively related to subjective well-being (H3), while job resources are positively related to subjective well-being (H4), were both supported. High job demands may result in a threat to workers' well-being (98), while providing teachers with some job resources could mitigate the negative effect caused by job demands (23). As for subjective well-being, some studies have revealed that it is beneficial for teachers to improve their subjective well-being through applying their character strengths (99, 100). In addition, the lack of time may contribute to reducing teachers' subjective well-being (93). However, in terms of job resources, according to the results, the more resources teachers are provided with, the higher subjective well-being they will feel. The supports from colleagues, school leaders, or students could help teachers maintain a high level of subjective well-being (93, 101), which was consistent with the present results.

#### The influence of subjective well-being on college teachers' online teaching anxiety

The results also found a negative correlation between college teachers' subjective well-being and online anxiety (H5 was supported). Subjective well-being is often considered as a positive psychological experience (102), and the higher teachers' subjective well-being, the less anxious they feel about online teaching. This finding corresponded to the research conducted by Zee and Koomen (103). When teachers have a great sense of subjective well-being, they are more flexible in dealing with the stress and anxiety they encounter in teaching (104). During online teaching, it is inevitable that teachers will encounter various problems, such as problems using technological devices, poor internet access, concern about students' online learning effectiveness, and so on (105), which will lead to teaching anxiety. However, according to the results of this study, it is helpful for college teachers to alleviate their teaching anxiety through improving their subjective well-being. As a previous literature review noted, there were positive correlations between teachers' sense of belonging, self-satisfaction, and subjective well-being (106). Therefore, in general, in order to alleviate teaching anxiety during online teaching, and to acquire good achievement and success in work as usual, it is vital to improve college teachers' subjective well-being through maintaining an intense sense of belonging (107), high job satisfaction (103), and a placid emotion.

#### The mediating influence of subjective well-being between job environment and online teaching anxiety

In this study, the role of subjective well-being in mediating between job environment and online teaching anxiety was confirmed (H4 was supported). Specifically, the effect of job demands on teachers' online teaching anxiety is influenced by their subjective well-being (H6 was supported); job resources can also influence teachers' online teaching anxiety through subjective well-being (H7 was supported). That is, in online teaching environments, teachers' subjective well-being is enhanced when they are provided with adequate job resources as well as being subject to lower external job demands, thereby alleviating their online teaching anxiety. Therefore, it is important to improve college teachers' subjective well-being and provide them with sufficient job resources while conducting online instruction. It is also a meaningful measure to improve college teachers' subjective well-being by creating a good online teaching job environment or condition. In addition, previous studies have shown that subjective well-being was a multi-angle construct, consisting of positive emotions, positive relationships, engagement, and so on (100). When teachers have positive emotions and positive relationships with their colleagues, administrators, and students, their subjective well-being will be improved. Therefore, in addition to providing the necessary online teaching hardware and equipment supports, the rich mental support and care from the school is also important for maintaining the high subjective well-being of teachers (108). Meanwhile, teachers can also maintain close contact with their colleagues, with whom they can share their happiness or difficulties in instruction or in their personal lives. This sharing will be helpful to improve their subjective well-being, so as to alleviate their online teaching anxiety.

## Conclusions

In recent years, online teaching has become an important instruction method at all stages of education. However, teachers also face some problems or anxiety during online teaching, and college teachers are no exception. What can college teachers do to mitigate their online teaching anxiety? Based on previous literature and studies, the current study attempted to respond to this question through exploring the influences of job environment and subjective well-being on college teachers' online teaching anxiety; meanwhile, the impact of job environment on subjective well-being was also explored.

The results of this study indicated that job demands, one factor of the job environment, were positively related to online teaching anxiety (H1), but were negatively related to subjective well-being (H4). Job resources, another factor of job environment, were negatively related to online teaching anxiety (H2), while being positively related to subjective well-being (H5). As for subjective well-being, it was negatively related to college teachers' online teaching anxiety (H3).

### Implications

This study theoretically firstly shows that the JD-R model can affect college teachers' online teaching anxiety. Specifically, there were negative relationships between job resources and online teaching anxiety, while job demands were also positively related to online teaching anxiety. Job demands can significantly positively predict teaching anxiety in the online education environment, which opens a new horizon for future research. What's more, in terms of the relationship between teachers' well-being and their teaching anxiety, past studies tended to focus on the exploration of the degree of teachers' well-being through the perspective of stress or burnout (109), but failed to identify the specific relationship between subjective well-being, one type of well-being, and online teaching anxiety. Furthermore, the JD-R model can also indirectly influence college teachers' online teaching anxiety through the mediating effects of subjective well-being. Therefore, the literature on college teachers' subjective well-being and online teaching anxiety has been enriched by the current study.

As for its practical implications, significant relationships between job environment, subjective well-being, and online teaching anxiety were supported through the exploration of this study. The current study provides some practical paths that can be applied to alleviate college teachers' teaching anxiety in online contexts. Firstly, regarding the relationship between job environment and online teaching anxiety, it is helpful for alleviating college teachers' online teaching anxiety to create a good online teaching job environment for teachers. Specifically, according to the results, job demands, one of the sub-dimensions of job environment, are positively related to online teaching anxiety, while the other subdimension, job resources, plays a negative role in creating online teaching anxiety. Thus, offering teachers sufficient job resources will be helpful for mitigating the teaching anxiety caused by job demands (23). What's more, according to the mediating effect of subjective well-being, teachers' online teaching anxiety can be relieved through improving teachers' subjective well-being by providing them with a good job environment in which there are sufficient job resources and lower job demands.

#### Limitations and future study

The current study mainly explored the influence of job environment and subjective well-being on college teachers' online teaching anxiety. However, in the future, there are still some other possible factors influencing teachers' teaching anxiety, such as gender, teaching experience, and teaching age, which can be determined in future studies. In addition, in the current study, the impact of subjective well-being, one of the important factors in the model, on online teaching anxiety and the influence it received from job demands and job resources were explored. However, it would also be worth investigating other forms of teachers' well-being such as cognitive well-being, health well-being, and social well-being in the model in future studies. Finally, teachers' online teaching anxiety is an important influencing factor of teaching quality and the academic performance of students, but this study lacks the relevant exploration of those predictors. Thus, the influence of teachers' teaching anxiety on teaching effectiveness and students' academic performance could be further explored in future studies.

## Data availability statement

The original contributions presented in the study are included in the article/supplementary material, further inquiries can be directed to the corresponding author/s.

## Ethics statement

The studies involving human participants were reviewed and approved by the Ethics Committee of Nanjing Normal University (NNU202208001). Written informed consent for participation was not required for this study in accordance with the national legislation and the institutional requirements.

## Author contributions

All authors contributed equally to the conception of the idea, implementing and analyzing the experimental results, writing the manuscript, and reading and approving the final version of the manuscript.

## Funding

Philosophy and Social Science Foundation of Jiangsu Province (CN) (No. 21ZXB007).

## Conflict of interest

The authors declare that the research was conducted in the absence of any commercial or financial relationships that could be construed as a potential conflict of interest.

## Publisher's note

All claims expressed in this article are solely those of the authors and do not necessarily represent those of their affiliated organizations, or those of the publisher, the editors and the reviewers. Any product that may be evaluated in this article, or claim that may be made by its manufacturer, is not guaranteed or endorsed by the publisher.
